# Micronutrition as a Therapeutic Strategy for Mitochondrial Dysfunction in Myalgic Encephalomyelitis/Chronic Fatigue Syndrome and Fibromyalgia: A Narrative Review

**DOI:** 10.3390/nu18162702

**Published:** 2026-08-19

**Authors:** Sergio Abanades, Irene Fernández, Nuria Capdevila, Francisco Cardona

**Affiliations:** 1 Centre Mèdic ISIC, 08008 Barcelona, Spain; 2 Department of Internal Medicine, Hospital Sant Pau, 08025 Barcelona, Spain; iferrnandez@santpau.cat; 3 Laboratorio LCN, Menac Medical Department, 08036 Barcelona, Spain; ncapdevila@laboratoriolcn.com; 4 Burrull Centro Médico Cardona, Spanish Society of Pulmonology and Thoracic Surgery (SEPAR), 08261 Barcelona, Spain; chesco@cmcbcn.com

**Keywords:** fibromyalgia, myalgic encephalomyelitis/chronic fatigue syndrome, ME/CFS, mitochondrial dysfunction, micronutrition, fatigue, NAD^+^, glutathione, precision nutrition

## Abstract

Fibromyalgia and myalgic encephalomyelitis/chronic fatigue syndrome (ME/CFS) are chronic multisystem disorders characterized by persistent fatigue, pain, cognitive dysfunction, sleep disturbances, and reduced quality of life. Increasing evidence implicates mitochondrial dysfunction, oxidative and nitrosative stress, immune dysregulation, and altered redox and nicotinamide adenine dinucleotide (NAD^+^) metabolism as interconnected mechanisms contributing to fatigue, although the strength of evidence varies across these pathways. Micronutrients are essential components of mitochondrial bioenergetics, antioxidant defense, and immune–metabolic regulation. This narrative review critically examines the mechanistic and clinical evidence supporting mitochondrial-oriented micronutritional interventions in fibromyalgia and ME/CFS, including NAD^+^ precursors, B-complex vitamins, magnesium, coenzyme Q10, alpha-lipoic acid, GlyNAC, L-carnitine, pyrroloquinoline quinone, taurine, and creatine. Mechanistic plausibility is distinguished from clinical efficacy, as disease-specific randomized controlled evidence remains limited for several interventions. We further discuss biomarkers, metabolic phenotyping, and precision nutrition within a systems-based micronutrition framework. Finally, we present the rationale for a future randomized, double-blind, placebo-controlled trial in fibromyalgia patients with clinically significant fatigue, which is planned for 2027, subject to ethics approval and prospective registration, as a strategy for future clinical validation.

## 1. Introduction

Fibromyalgia and myalgic encephalomyelitis/chronic fatigue syndrome (ME/CFS) are chronic, complex disorders characterized by persistent fatigue, pain, cognitive impairment, sleep disturbances, and substantial functional limitations. Although they represent distinct clinical entities, they share several symptoms and pathophysiological features, including fatigue, exercise intolerance, altered pain processing, and, in some patients, post-exertional symptom exacerbation [1]. A systematic review and meta-analysis demonstrated increased post-exercise pain in both ME/CFS and fibromyalgia, supporting clinically relevant overlap in responses to exertional stress [1].

Historically, fatigue in fibromyalgia and ME/CFS has frequently been interpreted predominantly within central or neurobehavioral frameworks. However, accumulating evidence indicates that fatigue and exercise intolerance in these disorders may also be associated with measurable abnormalities in cellular energy metabolism, redox homeostasis, immune–metabolic regulation, and responses to physiological stress [2,3,4,5]. In ME/CFS, alterations in mitochondrial bioenergetics and cellular metabolism have been reported using several experimental approaches [3,6,7,8]. In fibromyalgia, a growing body of evidence also supports alterations in mitochondrial function and quality control, although the extent to which these abnormalities represent primary disease mechanisms, secondary adaptations, or consequences of chronic illness remains uncertain [9,10,11]. Recent experimental evidence has additionally identified impaired mitochondrial biogenesis, dynamics, and quality control in skeletal muscle in a model of fibromyalgia [11].

These observations have contributed to increasing interest in mitochondria as potential contributors to fatigue pathophysiology. Mitochondria are not only responsible for much of cellular ATP production but also participate in redox regulation, calcium homeostasis, immune signaling, stress responses, apoptosis, and communication between cellular metabolic pathways. Nevertheless, evidence of mitochondrial abnormalities should not be interpreted as demonstrating that mitochondrial dysfunction is the sole or primary cause of either fibromyalgia or ME/CFS. Rather, mitochondrial alterations may represent one component of a broader and heterogeneous network involving immune, metabolic, neurological, and redox processes.

Micronutrients comprise vitamins, minerals, trace elements, and other nutritionally relevant molecules that participate in physiological processes as substrates, cofactors, coenzymes, antioxidants, or regulatory molecules. Mitochondrial metabolism is particularly dependent on adequate availability of multiple such factors because substrate oxidation, the tricarboxylic acid cycle, electron transport, redox reactions, and ATP synthesis require numerous interacting micronutrient-dependent enzymes and cofactors [12,13].

Within this framework, micronutrition can be conceptualized as an approach aimed at supporting physiological function through the coordinated optimization of nutritionally dependent metabolic pathways. Rather than following a pharmacological “single molecule–single target” paradigm, this approach recognizes that micronutrients operate within interconnected biochemical networks. Mitochondrial bioenergetics illustrates this interdependence particularly well: NAD^+^/NADH cycling, substrate oxidation, the tricarboxylic acid cycle, electron transport, glutathione-dependent redox regulation, and ATP production simultaneously depend on multiple nutritional factors [14,15].

Importantly, however, a strong physiological or mechanistic rationale does not necessarily imply demonstrated clinical efficacy. Evidence supporting individual mitochondrial-oriented micronutrients ranges from established biochemical functions and experimental studies to observational human data and, for a smaller number of interventions, randomized controlled trials. This distinction is particularly relevant in fibromyalgia and ME/CFS, where disease-specific clinical evidence remains limited and heterogeneous.

Accordingly, the objective of this narrative review is to critically examine the evidence linking mitochondrial dysfunction and altered redox–immune metabolism with fatigue in fibromyalgia and ME/CFS and to evaluate the mechanistic and clinical evidence supporting selected mitochondrial-oriented micronutritional interventions. Particular emphasis is placed on distinguishing biological plausibility from demonstrated clinical effects and on considering how systems-based micronutrition, biomarkers, and metabolic phenotyping could contribute to future research and more individualized therapeutic strategies.

## 2. Materials and Methods

This narrative review was conducted to synthesize and critically interpret current evidence regarding mitochondrial dysfunction and micronutritional therapeutic strategies in fibromyalgia and myalgic encephalomyelitis/chronic fatigue syndrome (ME/CFS). A structured, non-systematic search of PubMed/MEDLINE, Scopus, and the Cochrane Library was performed for English-language publications available up to 31 January 2026. Search strategies combined Medical Subject Headings (MeSH) terms and free-text keywords related to the following domains: “Fibromyalgia”, “Chronic Fatigue Syndrome”, “Myalgic Encephalomyelitis”, “ME/CFS”, “Mitochondrial Dysfunction”, “Mitochondrial Bioenergetics”, “Oxidative Stress”, “Nitrosative Stress”, “NAD”, “NAD+ Metabolism”, “NAD Precursors”, “Glutathione”, “Micronutrients”, “B-Complex Vitamins”, “Magnesium”, “Coenzyme Q10”, “Alpha-Lipoic Acid”, “Carnitine”, “Acetyl-L-Carnitine”, “Pyrroloquinoline Quinone”, “PQQ”, “Creatine”, “Taurine”, and “GlyNAC”. Boolean operators were used to refine combinations across pathophysiological and therapeutic domains.

The scope focused on studies addressing: (i) mitochondrial bioenergetics and metabolic dysfunction in fibromyalgia and ME/CFS; (ii) redox and immune–metabolic mechanisms contributing to fatigue; and (iii) clinical and translational evidence evaluating micronutritional interventions targeting mitochondrial pathways. Literature selection was guided by thematic relevance, methodological rigor, and contribution to the mechanistic or clinical questions addressed in this review. Priority was given to recent systematic reviews, randomized controlled trials, mechanistic and translational studies, and high-quality observational research. Particular consideration was given to human studies directly involving patients with fibromyalgia or ME/CFS and to randomized or placebo-controlled clinical trials when available. Preclinical studies were included when relevant to the mechanistic understanding of mitochondrial signaling, bioenergetics, redox regulation, or NAD^+^ metabolism. Clinical studies conducted in other populations were considered when disease-specific evidence was unavailable and when they provided relevant mechanistic or proof-of-concept information. Such evidence was interpreted as indirect and was not considered evidence of clinical efficacy in fibromyalgia or ME/CFS.

Additional relevant publications were identified through reference screening of key articles and citation tracking of influential reviews. Foundational studies were retained where necessary to support established biochemical mechanisms, whereas more recent studies were prioritized when they provided substantive updates to the evidence base.

Given the narrative nature of the review, no formal risk-of-bias assessment, quantitative meta-analysis, or GRADE certainty assessment was performed. Instead, the evidence was critically interpreted according to study design, population, methodological quality, direct relevance to fibromyalgia or ME/CFS, consistency of findings, and availability of randomized placebo-controlled evidence. Particular attention was given to distinguishing established biochemical and mechanistic functions from effects supported by clinical studies, and to avoiding extrapolation of findings from experimental models or other clinical populations as evidence of therapeutic efficacy in fibromyalgia or ME/CFS.

## 3. Pathophysiology of CFS/ME and Fibromyalgia

The pathophysiology of myalgic encephalomyelitis/chronic fatigue syndrome (ME/CFS) and fibromyalgia remains incompletely understood and is likely multifactorial. Although the two disorders are clinically distinct, overlapping abnormalities involving immune regulation, oxidative and nitrosative stress, cellular metabolism, and neuroendocrine signaling have been reported [16]. The strength and consistency of evidence for these mechanisms differ between the two conditions, and many findings remain associative rather than demonstrably causal. Accordingly, the mechanisms discussed below should be considered interacting components of heterogeneous disease processes rather than a single unified pathophysiological model.

### 3.1. Immune and Inflammatory Alterations

Immune dysregulation has been extensively investigated in ME/CFS, with studies reporting altered cytokine profiles, impaired natural killer (NK) cell function, and abnormalities in adaptive immune responses [17,18,19,20]. Reduced NK-cell cytotoxicity is among the more frequently reported immune abnormalities in ME/CFS, although findings across studies remain heterogeneous and no single immune alteration has been established as a diagnostic biomarker [18,20]. Evidence for immune and inflammatory alterations has also been reported in fibromyalgia, including changes in circulating cytokines and chemokines and evidence of low-grade inflammatory activation [21]. A systematic review and meta-analysis identified differences in several circulating cytokines in patients with fibromyalgia compared with healthy controls, while also demonstrating substantial heterogeneity across studies [21]. Specifically, pooled analyses identified higher concentrations of TNF-α, IL-6, IL-8, and IL-10, illustrating that the reported immune profile includes both pro- and anti-inflammatory signals rather than a uniform pro-inflammatory phenotype [21]. These findings support the presence of immune–inflammatory alterations in at least a subset of patients, although their magnitude and clinical relevance remain uncertain. It is also unclear whether these abnormalities represent primary disease mechanisms or secondary consequences of chronic pain, sleep disturbance, physical inactivity, or other associated factors.

In ME/CFS, abnormalities in immune responses to viral antigens, including Epstein–Barr virus (EBV), have also been described [19]. These observations have contributed to the hypothesis that persistent or dysregulated immune responses following infection may participate in disease pathophysiology in a subset of patients. However, current evidence does not establish persistent active viral infection as a universal mechanism of ME/CFS, and immune abnormalities are likely heterogeneous across patient populations. Collectively, chronic or dysregulated immune activation may interact with cellular metabolism and redox regulation, providing a potential mechanistic link between inflammatory signaling, oxidative stress, mitochondrial function, and fatigue [20].

### 3.2. Oxidative and Nitrosative Stress

Oxidative and nitrosative stress have been reported in both ME/CFS and fibromyalgia. Studies in ME/CFS have identified increased markers of oxidative damage and altered antioxidant defenses, with some associations between oxidative stress and symptom expression [22]. Reactive oxygen and nitrogen species (ROS/RNS) are physiologically involved in cellular signaling; however, excessive or sustained production can modify lipids, proteins, nucleic acids, and mitochondrial components [23]. Studies and reviews in fibromyalgia have likewise reported increased oxidative stress markers together with alterations in antioxidant defenses [24]. The latter literature supports an association between oxidative imbalance and fibromyalgia pathophysiology while not establishing oxidative stress as a primary causal mechanism. Mitochondria are simultaneously an important source and target of redox signaling, creating the potential for reciprocal interactions between impaired mitochondrial function and oxidative stress.

These findings provide a biologically plausible connection between redox imbalance and altered cellular bioenergetics in ME/CFS and fibromyalgia. Nevertheless, most available human studies are observational or cross-sectional and therefore cannot establish whether oxidative stress is a primary driver of disease, a consequence of altered metabolism and inflammation, or part of a bidirectional process. This distinction is relevant when considering antioxidant or mitochondrial-targeted interventions because normalization of a mechanistic biomarker does not necessarily predict improvement in clinical outcomes.

## 4. Mitochondrial Dysfunction as a Core Mechanism of Fatigue

### 4.1. Evidence from Bioenergetic Studies

Alterations in mitochondrial bioenergetics have been reported in ME/CFS using multiple experimental approaches. Studies have identified abnormalities in oxidative phosphorylation, respiratory capacity, mitochondrial membrane potential, and cellular ATP-related metabolism in immune cells and other cellular models [3,6,7,8]. However, findings are not entirely consistent across studies, and the specific mitochondrial abnormalities reported vary according to the cell type, experimental model, disease definition, and bioenergetic methodology used. Thus, current human evidence supports the presence of altered cellular energy metabolism in ME/CFS but does not establish a single, uniform mitochondrial defect as the primary cause of the disease.

Mitochondrial abnormalities have also been reported in fibromyalgia. Macchi et al. demonstrated impaired mitochondrial function in peripheral blood mononuclear cells (PBMCs) from patients with fibromyalgia, including a reduced Bioenergetic Health Index (BHI), with bioenergetic impairment correlating with clinical disease severity [9]. Structural mitochondrial abnormalities have additionally been identified by electron microscopy of PBMCs from patients with fibromyalgia [10]. Experimental evidence further suggests alterations in mitochondrial biogenesis, dynamics, and quality-control mechanisms in skeletal muscle models of fibromyalgia [11]. Importantly, the latter findings constitute mechanistic rather than direct clinical evidence and should not be interpreted as demonstrating equivalent abnormalities in human skeletal muscle.

Taken together, these studies support an association between mitochondrial alterations and both ME/CFS and fibromyalgia. Nevertheless, most available evidence is derived from relatively small observational, ex vivo, or experimental studies. Differences in patient selection, diagnostic criteria, cell populations, experimental conditions, and bioenergetic endpoints limit direct comparison between studies. Mitochondrial dysfunction should therefore be considered a plausible and potentially clinically relevant component of fatigue pathophysiology rather than an established universal causal mechanism.

### 4.2. The Redox–Immune–Mitochondrial Triangle

Mitochondrial function, redox homeostasis, and immune regulation are closely interconnected. Mitochondria are not only bioenergetic organelles but also signaling hubs involved in innate and adaptive immune responses, redox signaling, calcium regulation, and cellular stress responses [23,25]. Chronic or dysregulated immune activation can increase reactive oxygen and nitrogen species (ROS/RNS), while excessive oxidative stress can impair mitochondrial proteins, membranes, and nucleic acids. Conversely, mitochondrial dysfunction can amplify redox and inflammatory signaling, establishing potentially self-reinforcing interactions among these pathways [16,23,24,25].

Glutathione (GSH) is a major intracellular redox regulator, and disturbances in glutathione homeostasis can increase cellular susceptibility to oxidative stress [26]. NAD^+^ metabolism provides another potential connection between redox balance, energy metabolism, and cellular stress responses. NAD^+^ is required for cellular redox reactions and also serves as a substrate for enzymes including sirtuins and poly(ADP-ribose) polymerases (PARPs), thereby linking cellular energy status with DNA repair, stress responses, and mitochondrial regulation [27,28]. Oxidative DNA damage can activate PARPs, resulting in increased NAD^+^ consumption. Under conditions of excessive PARP activation, depletion of cellular NAD^+^ levels may compromise metabolic function and contribute to reductions in ATP availability [28,29]. This mechanism is well established at the cellular and experimental levels; however, its quantitative contribution to fatigue in patients with ME/CFS or fibromyalgia has not yet been established.

Immune cells, including T lymphocytes and NK cells, depend on metabolic reprogramming and mitochondrial function to support activation and effector functions. Consequently, alterations in cellular energy metabolism and redox signaling could contribute to impaired immune function and persistent inflammatory signaling. In ME/CFS and fibromyalgia, however, the proposed redox–immune–mitochondrial triangle should currently be regarded as an integrative mechanistic framework rather than a clinically validated causal model (Table 1).

### 4.3. Mitonuclear Communication and Retrograde Signaling

Beyond classical bioenergetic defects, mitochondrial dysfunction may influence disease-related cellular phenotypes through altered mitonuclear communication. Mitochondria continuously communicate with the nucleus through retrograde signaling pathways that respond to changes in ATP availability, ROS production, calcium homeostasis, NAD^+^ metabolism, and mitochondrial proteostasis. These signals can modify nuclear transcription and thereby regulate mitochondrial biogenesis, antioxidant responses, inflammation, and cellular adaptation to metabolic stress [30]. Experimental models demonstrate that mitochondrial stress can activate retrograde signaling pathways and reprogram nuclear gene expression, providing a plausible mechanism through which transient mitochondrial disturbances could generate more persistent metabolic and stress-response phenotypes [30]. In ME/CFS, metabolomic studies have identified patterns compatible with altered systemic energy metabolism and a hypometabolic-like state [3]. More recent analyses also support abnormalities in mitochondrial and metabolic pathways, although the relationship between these findings and specific mitonuclear signaling mechanisms remains incompletely established [31].

In fibromyalgia, mitochondrial functional and structural abnormalities have been identified in peripheral blood mononuclear cells (PBMCs) [9,10], while experimental models suggest altered mitochondrial quality-control pathways [11]. These observations provide biological plausibility for disturbed mitonuclear signaling. However, direct evidence demonstrating persistent pathological mitochondrial-to-nuclear transcriptional remodeling in patients with fibromyalgia remains limited. Accordingly, altered mitonuclear communication should currently be considered an emerging mechanistic hypothesis rather than an established disease mechanism.

Overall, mitochondrial dysfunction in ME/CFS and fibromyalgia appears to extend beyond ATP production alone and may involve reciprocal interactions among bioenergetic impairment, redox imbalance, immune signaling, and cellular stress responses. The relative contribution of these mechanisms is likely heterogeneous between patients and remains an important area for future mechanistic and biomarker-based research.

## 5. Micronutrients and Mitochondrial Bioenergetics

Micronutrients are required for virtually every step of mitochondrial energy metabolism. Subclinical insufficiencies or increased functional requirements associated with chronic inflammation and metabolic stress may create metabolic bottlenecks even in the absence of overt nutritional deficiency [12,15]. From a micronutritional perspective, these factors are considered components of interconnected physiological pathways rather than isolated pharmacological interventions. However, biochemical plausibility should be distinguished from demonstrated clinical efficacy. Recent reviews of supplementation in ME/CFS and nutraceutical interventions in fibromyalgia identified promising signals but also emphasized the substantial heterogeneity, small samples, and limited high-quality clinical evidence [32,33]. The principal mitochondrial roles and current levels of evidence are summarized in Table 2.

### 5.1. NAD^+^ Metabolism, NADH, and NAD^+^ Precursors

NAD^+^ and NADH form a central redox couple in cellular energy metabolism. NAD^+^ also serves as a substrate for PARPs and sirtuins, linking cellular redox status with DNA repair and mitochondrial regulation [27,28]. NAD^+^ availability can be supported through nutritional precursors including niacin, nicotinamide, nicotinamide riboside (NR), and nicotinamide mononucleotide (NMN). NR has demonstrated oral bioavailability and the capacity to increase NAD^+^ metabolism in humans [34]; however, disease-specific clinical evidence for NR or NMN in ME/CFS and fibromyalgia remains limited. In contrast, NADH has been evaluated directly in ME/CFS clinical trials, with potential benefits but inconsistent findings [35,36]. A recent systematic review similarly identified NADH and NADH–CoQ10 among interventions showing favorable fatigue signals while emphasizing methodological limitations [32]. The combined NADH–CoQ10 intervention is discussed below to avoid duplication.

### 5.2. B-Complex Vitamins and Magnesium

B-complex vitamins act as essential cofactors or coenzyme precursors in glycolysis, the tricarboxylic acid cycle, and mitochondrial energy metabolism [38]. A recent systematic review and meta-analysis in ME/CFS found no significant fatigue reduction when the quantitatively suitable RCTs were analyzed separately, although pooled eligible studies suggested a modest benefit; heterogeneity in formulations, treatment duration, and outcome measures limits firm conclusions [39].

Magnesium is required for ATP-dependent processes and numerous enzymatic reactions involved in cellular energetics [40]. In fibromyalgia, a randomized, double-blind, placebo-controlled trial reported improvements in pain and stress-related outcomes in selected patients, although effects on fatigue, sleep, and quality of life were not significant [41]. These findings support further investigation of B vitamins and magnesium within a metabolic and micronutritional framework while highlighting the need for better-defined populations and supplementation strategies.

### 5.3. Coenzyme Q10

Coenzyme Q10 (CoQ10) is an essential component of the mitochondrial electron transport chain and also functions as a lipophilic antioxidant. Alterations in CoQ10 status have been reported in fatigue-related disorders, including ME/CFS [42]. CoQ10 has comparatively more direct disease-specific clinical evidence than several other mitochondrial-oriented micronutrients. In a randomized, double-blind, placebo-controlled trial involving patients with ME/CFS, combined supplementation with CoQ10 and NADH improved several fatigue-related and health-related quality-of-life outcomes [37]. This combination is also among the interventions identified as potentially beneficial in a recent systematic review [32]. However, because CoQ10 and NADH were administered together, their individual contributions cannot be established.

### 5.4. Alpha-Lipoic Acid

Alpha-lipoic acid (ALA) is a mitochondrial dehydrogenase cofactor with antioxidant and redox-regulating properties [43,44]. Its effects in diabetic neuropathy and experimental models provide a rationale for its investigation in fibromyalgia [45]. However, a small randomized, double-blind, placebo-controlled crossover trial in fibromyalgia patients found no significant improvement in pain with ALA, despite dose escalation up to 1800 mg/day [46]. Interpretation remains limited by the small sample, short treatment duration, and potential influence of dose, formulation, R/S-isomer composition, and patient phenotype on biological response. A multi-component supplement containing ALA, CoQ10, magnesium, and other micronutrients showed some improvement in pain, although the contribution of ALA cannot be isolated [47]. Overall, ALA has strong mechanistic plausibility, but disease-specific clinical efficacy remains unconfirmed.

### 5.5. Glutathione Repletion and GlyNAC

Glutathione (GSH) is a major intracellular antioxidant and an important regulator of mitochondrial redox homeostasis [26]. Disturbances in oxidative balance have been reported in both ME/CFS and fibromyalgia [22,24], providing a rationale for strategies aimed at supporting glutathione synthesis. However, whether restoration of glutathione status translates into clinically meaningful improvements in fatigue remains insufficiently established in these populations.

#### N-Acetylcysteine and Glycine

N-acetylcysteine (NAC) provides cysteine for glutathione synthesis, while glycine is another required substrate. Combined supplementation with glycine and NAC (GlyNAC) has been investigated as a strategy to restore glutathione availability and improve mitochondrial and redox function. Human studies, including a randomized placebo-controlled trial in older adults, have reported improvements in glutathione status, oxidative stress, mitochondrial function, inflammation, and physical function [48,49]. However, these findings derive primarily from aging populations rather than ME/CFS or fibromyalgia patients and therefore represent translational proof-of-concept rather than disease-specific evidence of efficacy.

### 5.6. Additional Mitochondrial-Supporting Micronutrients

#### 5.6.1. Acetyl L-Carnitine

Carnitine facilitates the transport of long-chain fatty acids into mitochondria for β-oxidation and therefore plays an important role in mitochondrial energy metabolism [50]. An exploratory randomized study comparing acetyl-L-carnitine and propionyl-L-carnitine in ME/CFS reported improvements in fatigue-related outcomes, although the absence of a placebo group and limited replication restrict the strength of the evidence [51]. A recent systematic review also identified carnitine-containing interventions among nutritional strategies showing potentially favorable signals in ME/CFS while emphasizing the overall low certainty and heterogeneity of the evidence [32].

#### 5.6.2. Pyrroloquinoline Quinone (PQQ), Taurine, and Creatine

Pyrroloquinoline quinone (PQQ) has been associated with mitochondrial biogenesis and regulation of PGC-1α-related signaling [52,53,54]. These properties provide a mechanistic rationale for mitochondrial support, but disease-specific clinical evidence in ME/CFS or fibromyalgia remains insufficient. Taurine contributes to mitochondrial function, membrane stability, and calcium homeostasis, although clinical intervention evidence in these conditions is similarly limited [55].

Creatine acts through the phosphocreatine system as an intracellular energy buffer, facilitating rapid ATP regeneration in tissues with high energetic demands [56]. In a randomized, double-blind, placebo-controlled trial in women with fibromyalgia, creatine increased intramuscular phosphorylcreatine and improved muscle strength, but did not significantly improve pain, cognitive function, sleep, or quality of life [57]. Thus, creatine illustrates the distinction between demonstrated effects on muscle bioenergetics and broader clinical efficacy.

#### 5.6.3. Synergistic Micronutrient Combinations

The micronutritional approach considered in this review differs conceptually from treating micronutrients as isolated pharmacological agents. Mitochondrial energy metabolism depends on an interconnected network of substrates, cofactors, redox systems, and signaling pathways; consequently, functional limitations in multiple components may collectively constrain mitochondrial function. Recent reviews of nutritional interventions in ME/CFS and fibromyalgia identified promising signals but also substantial heterogeneity in formulations, populations, and clinical outcomes [32,33] (Table 3). Accordingly, biological plausibility for coordinated metabolic support should not be interpreted as evidence that a fixed micronutrient combination will benefit all patients. A systems-based micronutritional approach may therefore be integrated within a precision-nutrition framework, considering nutritional status, metabolic phenotype, disease severity, comorbidities, and potentially mitochondrial or redox biomarkers when selecting interventions. At present, however, validated biomarkers capable of reliably predicting individual responses are lacking. Prospective studies combining clinical phenotyping, objective metabolic biomarkers, and adequately powered placebo-controlled designs are needed to determine whether targeted multi-micronutrient strategies provide advantages over isolated supplementation.

## 6. Proposal for a Randomized Placebo-Controlled Trial in Fibromyalgia

Fatigue remains one of the most disabling and insufficiently treated symptoms of fibromyalgia. Based on evidence linking mitochondrial dysfunction with disease severity and fatigue-related mechanisms in fibromyalgia [9,10,11], together with the clinical and mechanistic evidence reviewed above, a randomized, double-blind, placebo-controlled trial is planned to evaluate a systems-based micronutritional intervention in patients with fibromyalgia and severe fatigue. The study is intended as a prospective validation of the hypothesis developed in this review, rather than as evidence supporting the efficacy of the proposed intervention. The trial is currently in the planning stage and is expected to begin in early 2027. At the time of preparation of this manuscript, the study has not yet commenced recruitment and has not received final ethics committee approval or clinical trial registration. These details will be obtained and publicly reported before participant recruitment begins.

### 6.1. Study Population

The planned study will include adults with a confirmed diagnosis of fibromyalgia according to established diagnostic criteria and a PROMIS Fatigue T-score > 60, thereby selecting a population with clinically significant fatigue and a substantial unmet therapeutic need.

### 6.2. Intervention Rationale

**Figure 1:**
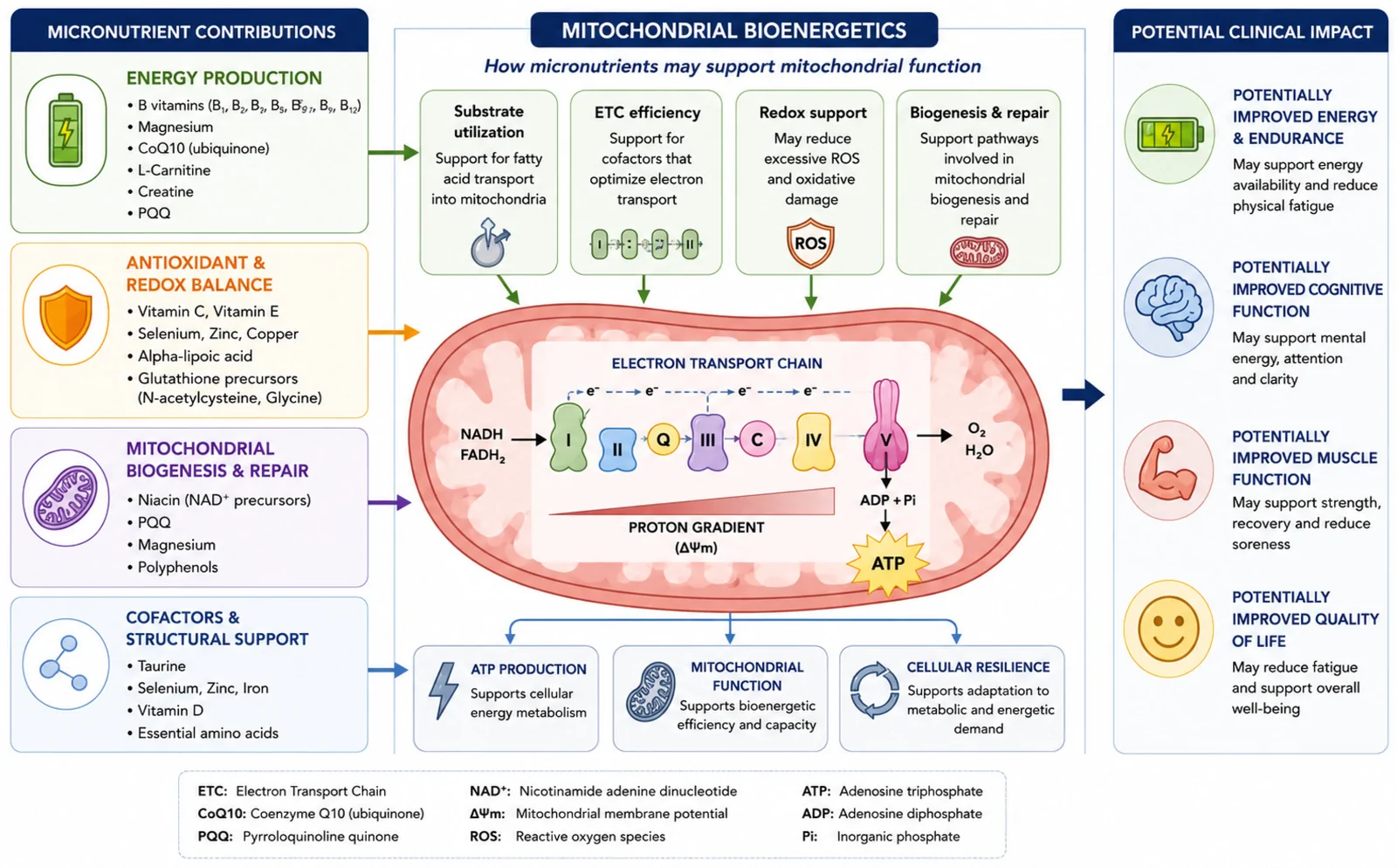
Proposed Interactions between Micronutrients, Mitochondrial Bioenergetics, and Fatigue-Related Outcomes.

The investigational formulation is designed according to the systems-based micronutritional framework developed in this review. Rather than targeting a single pathway, it aims to provide coordinated support for glutathione and redox homeostasis (GlyNAC), NAD^+^ metabolism/precursors, electron transport and mitochondrial bioenergetics (CoQ10 and B-complex vitamins), and ATP production and buffering (magnesium, carnitine, creatine, and alpha-lipoic acid) (Figure 1). The composition is based primarily on physiological and mechanistic complementarity rather than on the assumption that each component has independently demonstrated efficacy in fibromyalgia. As summarized in Section 5, the level of clinical evidence differs substantially among individual components. The proposed placebo-controlled trial is therefore intended to determine whether their coordinated administration translates into clinically meaningful improvements in fatigue.

## 7. Conclusions

Mitochondrial dysfunction, redox imbalance, and immune–metabolic dysregulation may contribute to fatigue in fibromyalgia and ME/CFS. Micronutrients support mitochondrial bioenergetics and redox homeostasis, although clinical evidence varies substantially among interventions. A systems-based micronutritional approach is biologically plausible but remains complementary and investigational rather than an established therapy. Current evidence is limited by heterogeneous studies and generally low-to-moderate certainty. Well-designed randomized placebo-controlled trials are needed to determine whether coordinated micronutritional strategies can meaningfully improve fatigue and related outcomes.

## Figures and Tables

**Table 1:** The Redox–Immune–Mitochondrial Triangle in Fatigue Pathophysiology.

Component	Main Alteration	Potential Consequence	Evidence
Mitochondria	Impaired oxidative phosphorylation and reduced energetic reserve	Reduced ATP availability and fatigue	Human and mechanistic evidence [3,6,7,8,9,10,11]
Redox balance	Increased ROS/RNS and impaired antioxidant defenses	Oxidative damage and further mitochondrial dysfunction	Human observational and mechanistic evidence [22,23,24,26]
Immune system	Altered cytokine signaling and NK/T-cell dysfunction	Persistent inflammatory and metabolic stress	Human observational/meta-analytic evidence [17,18,19,20,21]
Integrated interaction	Reciprocal amplification of mitochondrial, redox, and immune abnormalities	Persistent fatigue and reduced resilience to physiological stress	Integrative mechanistic framework; causality not established

**Table 2:** Key Micronutrients Involved in Mitochondrial Function and Fatigue.

Micronutrient	Primary Mitochondrial Role	Potential Relevance to Fatigue	Current Evidence
NAD^+^ precursors (niacin, nicotinamide, NR, NMN)	NAD^+^ synthesis; redox and mitochondrial regulation	Supports metabolic flexibility and mitochondrial homeostasis	Strong mechanistic evidence; limited disease-specific clinical evidence [27,28,34]
NADH	Redox cofactor; electron transfer and oxidative phosphorylation	Supports mitochondrial ATP production	Limited disease-specific clinical trial evidence [32,35,36,37]
B-complex vitamins	Cofactors in glycolysis, TCA cycle, and ETC	Supports energy metabolism and prevents metabolic bottlenecks	Established biochemical role; limited disease-specific clinical evidence [32,38,39]
Magnesium	ATP stabilization; enzymatic cofactor	Supports cellular energetics and neuromuscular function	Mechanistic + disease-specific RCT evidence [40,41]
Coenzyme Q10	ETC electron transfer; antioxidant	Supports ATP synthesis and redox homeostasis	Mechanistic + disease-specific clinical evidence [32,37,42]
Alpha-lipoic acid	Mitochondrial dehydrogenase cofactor; redox regulation	Supports oxidative metabolism and antioxidant defenses	Strong mechanistic evidence; fibromyalgia RCT showed negative results [43,44,45,46,47]
NAC + glycine (GlyNAC)	Glutathione synthesis	Supports mitochondrial redox homeostasis	Mechanistic + human RCT evidence outside of ME/CFS/fibromyalgia [26,48,49]
Acetyl-L-carnitine	Fatty-acid transport and β-oxidation	Supports mitochondrial energy production	Limited disease-specific clinical evidence [32,50,51]
PQQ	Mitochondrial biogenesis and signaling	May support mitochondrial quantity and efficiency	Predominantly mechanistic/preclinical evidence [52,53,54]
Taurine	Mitochondrial membrane and calcium homeostasis	Supports mitochondrial and neuromuscular function	Predominantly mechanistic evidence [55]
Creatine	Phosphocreatine energy buffering	Facilitates rapid ATP regeneration	Disease-specific RCT evidence in fibromyalgia [56,57]

**Table 3:** Summary of Clinical Evidence for Mitochondrial-Oriented Micronutrients in Fatigue.

Intervention	Population	Study Design	Main Findings	Evidence/Limitations
NADH	ME/CFS	Placebo-controlled clinical trials	Potential improvement in fatigue-related outcomes	Direct evidence, but small studies and variable findings [32,35,36]
CoQ10 + NADH	ME/CFS	Double-blind, placebo-controlled RCT	Improvements in fatigue-related and QoL outcomes	Combination prevents attribution to either component individually [32,37]
Magnesium	Fibromyalgia	Double-blind, placebo-controlled RCT	Improvements in pain and stress-related outcomes	No significant improvement in fatigue, sleep, or QoL [41]
ALA	Fibromyalgia	Double-blind, placebo-controlled crossover RCT	No significant improvement in pain or major clinical outcomes	Small sample; short duration; dose/formulation and phenotype may influence response [46]
GlyNAC	Older adults	Placebo-controlled RCT	Improved GSH status, oxidative stress, mitochondrial and physical-function measures	Promising translational evidence, but not disease-specific [48,49]
Acetyl-L-carnitine	ME/CFS	Exploratory randomized study	Potential improvement in fatigue	No placebo group; small study and limited replication [32,51]
Creatine	Fibromyalgia	Double-blind, placebo-controlled RCT	Increased muscle phosphocreatine and muscle strength	No significant improvement across broader clinical outcomes [57]

## Data Availability

No new data were created or analyzed in this study. Data sharing is not applicable to this article.

## Abbreviations

The following abbreviations are used in this manuscript:

**Abbreviation**	**Definition**
ME/CFS	Myalgic encephalomyelitis/chronic fatigue syndrome
NAD^+^	Nicotinamide adenine dinucleotide
NADH	Reduced nicotinamide adenine dinucleotide
ATP	Adenosine triphosphate
ROS	Reactive oxygen species
PBMCs	Peripheral blood mononuclear cells
CoQ10	Coenzyme Q10 (ubiquinone)
ALA	Alpha-lipoic acid
NAC	N-acetylcysteine
GlyNAC	Glycine plus N-acetylcysteine
GSH	Glutathione
NR	Nicotinamide riboside
NMN	Nicotinamide mononucleotide
PQQ	Pyrroloquinoline quinone
TCA	Tricarboxylic acid cycle
ETC	Electron transport chain
RCT	Randomized controlled trial
QoL	Quality of life
PROMIS	Patient-Reported Outcomes Measurement Information System
